# Ideal Cardiovascular Health Metrics and Incidence of Ischemic Stroke Among Hypertensive Patients: A Prospective Cohort Study

**DOI:** 10.3389/fcvm.2020.590809

**Published:** 2020-11-23

**Authors:** Yuchen Ying, Shaoyi Lin, Fanqian Kong, Yuying Li, Shujun Xu, Xiaofeng Liang, Changyi Wang, Liyuan Han

**Affiliations:** ^1^Zhejiang Provincial Key Laboratory of Pathophysiology, School of Medicine, Ningbo University, Ningbo, China; ^2^Department of Elderly Health Care and Management, School of Health Services and Management, Ningbo College of Health Sciences, Ningbo, China; ^3^Cardiology Department, Ningbo First Hospital, Ningbo, China; ^4^Department of Medical Record and Statistics, Ningbo Medical Center Lihuili Hospital, Ningbo, China; ^5^Shenzhen Polytechnic, Shenzhen, China; ^6^Chinese Preventive Medicine Association, Beijing, China; ^7^Department of Chronic Disease Prevention and Control, Shenzhen Nanshan Center for Chronic Disease Control, Shenzhen, China; ^8^Hwa Mei Hospital, University of Chinese Academy of Sciences, Ningbo, China; ^9^Department of Global Health, Ningbo Institute of Life and Health Industry, University of Chinese Academy of Sciences, Ningbo, China

**Keywords:** ideal cardiovascular health metrics, ischemic stroke, hypertensive patients, prospective study, hyperhomocysteinemia

## Abstract

**Background:** This study aimed to assess the relationship between ideal cardiovascular health (CVH) metrics and incident ischemic stroke (IS) in hypertensive patients, especially those with hyperhomocysteinemia (HHcy).

**Methods:** A prospective cohort study enrolled 5,488 hypertensive patients in Nanshan District of Shenzhen City in southern China from September 2011 to December 2017. CVH metrics were defined according to the American Heart Association. Cox proportional hazards models were used to examine the associations between the number of ideal CVH metrics and the incidence of IS by calculating multivariable-adjusted hazard ratios (HRs) and 95% CI.

**Results:** During an average follow-up of 5.7 years, 340 IS patients were identified. Compared with those having 0 ideal CVH metrics, the HRs (95% CIs) for IS among those with 1, 2, 3, 4, and 5–6 ideal CVH metrics were 0.62 (0.31–1.25), 0.37 (0.19–0.74), 0.37 (0.18–0.74), 0.34 (0.16–0.71), and 0.28 (0.12–0.63), respectively (*P* < 0.001). An ideal healthy diet score and ideal fasting blood glucose level were independently associated with IS among participants, with HRs (95% CIs) of 0.53 (0.33–0.86) and 0.32 (0.17–0.66), respectively. Additionally, compared with those with normal total homocysteine (tHcy) levels (<15 μmol/L), the HR (95% CI) for IS among participants with HHcy and who had 5–6 ideal CVH metrics was 0.50 (0.27–0.92).

**Conclusion:** An increased number of ideal CVH metrics was inversely associated with the incidence of IS in hypertensive patients. The participants with HHcy who had 5–6 ideal CVH metrics exhibited a lower IS risk than those with normal tHcy levels.

## Introduction

Stroke is the leading cause of mortality and disability among adults in China (1) and 87% of stroke-related deaths are caused by ischemic stroke (IS) (2). Evidence suggests that hypertension and hyperhomocysteinemia (HHcy) have a synergistic effect on the risk of IS (3). We previously found that the hazard ratio (HR) (95% CI) of IS caused by HHcy was 2.18 (1.65–2.89) in a hypertensive Chinese population (3). Importantly, ~80.3% of the hypertensive patients in this Chinese sample had HHcy (4), and the risk of stroke faced by patients with HHcy was 12.7 times higher than that of adults without hypertension and HHcy (5).

The American Heart Association (AHA) has defined ideal cardiovascular health (CVH) metrics. These include four health behaviors [not smoking, a body mass index [BMI] < 24 kg/m^2^, physical activity at goal levels, and a healthy diet composition], and three health factors (untreated total cholesterol <11.1 mmol/L, untreated blood pressure <130/80 mmHg, and untreated fasting blood glucose <5.5 mmol/L). Mounting evidence shows an inverse association between the number of ideal CVH metrics and the incidence of stroke across diverse populations and types of patients (6, 7). However, there are limited data to inform ideal CVH metrics that align with the AHA's definition of IS incidence in hypertensive patients, and especially in patients with both hypertension and HHcy. We hypothesized that an increased number of ideal CVH metrics would be associated with a decreased incidence of IS in hypertensive patients, and especially those with HHcy. Therefore, we performed a prospective cohort study to examine whether ideal CVH metrics were associated with a lower risk of IS among Chinese hypertensive patients.

## Methods

### Study Population

We performed a community-based, observational, long-term follow-up study to investigate the epidemiology of IS in Chinese hypertensive adults (3). This type of study design has been described previously (3). This prospective study was based on data from the hypertension management information systems of 60 community health service centers (CHSCs) in Nanshan District of Shenzhen City in southern China. The inclusion criteria were as follows: (1) patients who had essential hypertension and were ≥20 years of age; (2) local residents who had lived in Shenzhen for ≥6 months; (3) Chinese citizens who could receive follow-up without difficulty for at least 3 years; and (4) patients whose health records had been established in a CHSC hypertension management information system. The exclusion criteria were as follows: (1) patients with secondary hypertension, cancer, severe liver or kidney disease, or pregnancy; and (2) hypertensive patients with IS.

From April 2010 to September 2011, 5,935 hypertensive patients aged ≥20 years and who had lived in Shenzhen for ≥6 months completed the baseline survey (Supplementary Figure 1). All of the patients were Chinese citizens who could be followed up without difficulty for at least 3 years, and had established health records at the CHSCs. The study was performed according to the guidelines of the Helsinki Declaration and was approved by the Ethics Committee of the Shenzhen Nanshan Center for Chronic Disease Control. Signed informed consent was obtained from all of the participants.

### Data Collection

During the baseline survey, we used a validated questionnaire to collect demographic characteristics of the participants. For more details about characteristics, please see Supplementary Table 1.

In addition to the survey items, physical examination data including height and weight were measured at baseline. Height without shoes was measured to the nearest 1 cm with a standard right-angle device. Weight was measured to the nearest 0.1 kg with a spring balance. BMI was calculated as weight/height^2^ (kg/m^2^). Blood pressure was measured three times at 30-s intervals, using a standardized automatic electronic sphygmomanometer after 5–10 min of rest with the participants in a seated posture, and the average of the three measurements was used in all of the analyses. Essential hypertension was diagnosed based on systolic blood pressure (SBP) ≥ 140 mmHg or diastolic blood pressure (DBP) ≥ 90 mmHg, or self-reported use of antihypertensive medication (8).

### Laboratory Methods

Blood samples were collected from the antecubital vein in the morning after an overnight fast. Serum uric acid levels were measured by quantitative determination with uricase, plasma total homocysteine (tHcy) levels were measured by the circulating enzyme method, and serum creatinine levels were determined by the Jaffe method using an automatic biochemical analyzer (Hitachi 7080). tHcy levels ≥15 μmol/L was defined as HHcy, according to the Guidelines for Prevention and Treatment of Hypertension in China (2018 Revised Edition) (9). Total cholesterol (TC) and fasting blood glucose (FBG) were measured enzymatically with an autoanalyzer (Hitachi 7080).

### Assessment of Ideal CVH Metrics

The CVH metrics adopted in this study were consistent with the CVH metrics proposed by the AHA, which include seven health behaviors and factors: healthiness of diet, blood pressure (BP), physical activity, BMI, smoking status, TC, and FBG (10). According to the AHA guidelines (10), CVH metrics are classified as ideal, intermediate, or poor. The details of ideal, intermediate, and poor CVH metrics as defined by the AHA and by our modified version are described in Supplementary Table 2. Because all of the subjects in the study were hypertensive patients, BP was not included.

A total CVH score was calculated by summing scores for each of the 6 CVH metrics, ranging from 0 to 6, with the highest score indicating a better CVH. Total CVH score was then categorized into grades 0, 1, 2, 3, 4, 5, and 6.

### Outcome Assessment

December 2017 was the end point of the follow-up period. After 5.7 years of follow-up, face-to-face interviews, physical exams, and biochemical measurements were performed at the Shenzhen Nanshan Center for Chronic Disease Control. The methods were consistent with those in the baseline survey.

The primary outcome was the first occurrence of IS. This outcome was confirmed by examination of medical records including reports of symptoms and examination results. The examinations included computed tomography (CT), magnetic resonance imaging (MRI), cerebral angiography, and transcranial doppler ultrasound, in agreement with World Health Organization (WHO) criteria (11), and were conducted by two cerebrovascular experts.

Transient ischemic attack (TIA) was also diagnosed as IS if the scan did not visualize an infarction or hemorrhage, but the patient had symptoms that met the WHO criteria for IS (12). Both stroke and TIA were defined as cerebralvascular accident (CVA) (13).

IS was coded as I63 and I64 according to the 10th Revision of the International Classification of Diseases (ICD-10) and classified IS into 5 subtypes: large artery atherosclerosis, cardioembolism, small-vessel occlusion, stroke of other cause, and stroke of undetermined cause according to the TOAST classification (14).

### Statistical Analysis

Categorical variables were described as percentages (%) and were compared using the chi-square test. Continuous variables were described as mean ± SD and were compared using one-way analysis of variance. Variables with skewed distributions were log-transformed to normal distributions.

Person years of follow-up were calculated from the date of the initial baseline interview until the date when the participants were diagnosed with IS, the date of death, or the end of the follow-up period, whichever occurred first.

Multivariable Cox proportional hazards regression models were used to examine the association between the number of baseline ideal CVH metrics and the incidence of IS by calculating HRs and 95% CIs. Because only 35 (0.75%) participants had the highest CVH score of 6, we combined them with the participants who had a CVH score of five. We fitted three models to the data. Model 0 (M0) was not adjusted. Model 1 (M1) was adjusted for age and sex. Model 2 (M2) was further adjusted for education level, alcohol consumption, depression, years of hypertension, antihypertensive drug use, diabetes, family history of stroke, SBP, DBP, low density lipoprotein (LDL), uric acid (UA), triglyceride (TG), tHcy, and creatinine.

We tested the interactions between CVH metrics and age (<60 years vs. ≥60 years), sex, hypertension duration (<5 years vs. ≥ 5 years), BP (<130/80 mmHg vs. ≥130/80 mmHg) and tHcy (<15 μmol/L vs. ≥15 μmol/L) after controlling for the aforementioned covariates.

We also analyzed the risk of IS events, according to the combined ideal CVH metrics, among participants with HHcy compared with participants with normal tHcy levels. To test the *P* trend, we assigned a numerical value to the number of ideal CVH metrics and analyzed it as a continuous variable using the PHREG process in SAS.

The data were analyzed using SAS 9.3 (SAS Institute, Cary, NC). Two-sided *p* < 0.05 were considered statistically significant.

## Results

### Baseline Characteristics

This prospective study initially enrolled 5,935 participants, of whom 447 who presented with IS and CHD were excluded (Supplementary Figure 1). After an average follow-up period of 5.7 years, 826 of the remaining 5,488 participants were lost to follow-up (15.05%), 348 refused to continue, 206 returned to their hometowns, 164 changed their telephone numbers, 73 left the country, and 35 died. Finally, 4,662 participants were successfully followed and included in the prospective study.

Supplementary Table 1 presents the baseline characteristics of the participants according to the number of ideal CVH metrics. The mean (SD) age of the study participants was 41.3 (7.4) years, and 49.9% of the participants were male. Of the 4,662 participants, only 0.75% *(n* = 35) had 6 ideal CVH metrics; most of them (57.61%, *n* = 2,686) had 3–4 ideal CVH metrics. The participants with a greater number of ideal CVH metrics were more likely to be female, to be younger, to have higher education, to consume less alcohol, and to have experienced hypertension for only 2–5 years.

### Comparison of Baseline Characteristics Between Participants Lost and Not Lost to Follow-Up

Supplementary Table 3 summarizes the characteristics of the participants lost and not lost to follow-up. Our analysis showed that fruit intake, age, and SBP differed significantly between the two groups (all *P* < 0.05).

### Association Between the Number of Ideal CVH Metrics and Incident Ischemic Stroke Risk

After an average follow-up period of 5.7 years, a total of 340 IS cases were identified. Unadjusted and adjusted HRs (95% CIs) of the incidence of IS by both CVH metrics and sex are presented in Table 1. Multivariable adjusted Cox proportional hazards models indicated that the number of ideal CVH metrics were inversely associated with the incidence of IS after controlling for the aforementioned covariates. Compared with the participants with 0 ideal CVH metrics, the HRs (95% CIs) for the participants with scores of 5–6 ideal CVH metrics among all of the participants was 0.28 (0.12–0.63). For males, it was 0.22 (0.06–0.81), and for females it was 0.25 (0.07–0.86).

**Table 1:** The HRs (95% CIs) of incident ischemic stroke by Cardiovascular Health Metrics and sex.

Characteristics^*^	**Cardiovascular health metrics**						***P* trend**
	**0**	**1**	**2**	**3**	**4**	**5–6**	
**MALES**							
Cases	8	23	47	42	21	7	
Number of participants	84	348	647	700	401	147	
M0^†^	1 [Reference]	0.73 (0.32–1.64)	0.35 (0.16–0.75)	0.41 (0.19–0.88)	0.37 (0.16–0.85)	0.22 (0.07–0.64)	0.006
M1^‡^	1 [Reference]	0.70 (0.31–1.58)	0.32 (0.15–0.70)	0.38 (0.18–0.83)	0.34 (0.15–0.79)	0.21 (0.07–0.63)	0.005
M2^§^	1 [Reference]	0.80 (031–2.07)	0.37 (0.14–0.94)	0.45 (0.17–1.17)	0.37 (0.12–1.08)	0.22 (0.06–0.81)	0.019
**FEMALES**							
Cases	4	31	52	60	34	11	
Number of participants	38	284	598	741	506	168	
M0^†^	1 [Reference]	0.47 (0.16–1.36)	0.42 (0.15–1.17)	0.35 (0.12–0.99)	0.33 (0.11–0.95)	0.31 (0.10–1.00)	0.036
M1^‡^	1 [Reference]	0.47 (0.16–1.36)	0.41 (0.14–1.14)	0.35 (0.12–0.98)	0.32 (0.11–0.93)	0.31 (0.09–1.00)	0.033
M2^§^	1 [Reference]	0.42 (0.14–1.29)	0.30 (0.10–0.88)	0.25 (0.08–0.77)	0.25 (0.08–0.75)	0.25 (0.07–0.86)	0.020
**TOTAL**							
Cases	12	54	99	102	55	18	
Number of participants	122	632	1,245	1,441	907	315	
M0^†^	1 [Reference]	0.57 (0.30–1.07)	0.39 (0.21–0.71)	0.37 (0.20–0.69)	0.36 (0.19–0.67)	0.28 (0.13–0.59)	<0.001
M1^‡^	1 [Reference]	0.55 (0.29–1.04)	0.37 (0.20–0.68)	0.36 (0.19–0.66)	0.34 (0.17–0.64)	0.27 (0.13–0.58)	<0.001
M2^§^	1 [Reference]	0.62 (0.31–1.25)	0.37 (0.19–0.74)	0.37 (0.18–0.74)	0.34 (0.16–0.71)	0.28 (0.12–0.63)	<0.001

*BMI, body mass index*.

* *All non-pregnant participants older than 20 years with available cardiovascular health metrics were included*.

† *Not adjusted*.

‡ *Adjusted for age, sex*.

§ *Adjusted for age, sex, education, alcohol consumption, diabetes mellitus, depression, family history of stroke, years of hypertension, antihypertensive medication, low density lipoprotein, systolic blood pressure, diastolic blood pressure, uric acid, triglyceride, total homocysteine and creatinine*.

The Kaplan-Meier curves presented in Supplementary Figure 2 indicate that a graded decrease in the risk of IS is associated with an increased number of ideal CVH metrics (log-rank *P* = 0.002).

The associations between each CVH metric and IS risk are shown in Table 2. After adjustments for the aforementioned covariates, the ideal healthy diet score (7–9 points) was independently associated with a decreased risk of incident IS, with a HR (95% CI) of 0.53 (0.33–0.86). The ideal FBG (<5.5 mmol/L) was also associated with a decreased risk of incident IS, with a HR (95% CI) of 0.32 (0.17–0.62).

**Table 2:** The HRs (95% CIs) of incident ischemic stroke by each cardiovascular health metrics.

**Characteristics**	**β**	HR^*^	**95% *CI***	***P***
Smoking status				
Ideal	−0.13	0.88	0.60–1.29	0.523
Intermediate	0.08	1.08	0.69–1.67	0.727
Poor	Reference			
physical activity				
Ideal	−0.12	0.88	0.67–1.16	0.371
Intermediate	0.08	1.08	0.69–1.68	0.733
Poor	Reference			
BMI				
Ideal	−0.17	0.84	0.58–1.22	0.375
Intermediate	−0.16	0.85	0.59–1.21	0.380
Poor	Reference			
Healthy diet score				
Ideal	−0.63	0.53	0.33–0.86	0.010
Intermediate	−0.43	0.65	0.42–0.99	0.047
Poor	Reference			
Total serum cholesterol				
Ideal	0.05	1.05	0.62–1.76	0.861
Intermediate	−0.06	0.94	0.63–1.41	0.771
Poor	Reference			
Fasting blood glucose				
Ideal	−1.12	0.32	0.17–0.62	<0.001
Intermediate	−0.94	0.39	0.23–0.66	<0.001
Poor	Reference			

*BMI, body mass index*.

* *Adjusted for age, sex, education, alcohol consumption, diabetes mellitus, depression, family history of stroke, years of hypertension, antihypertensive medication, low density lipoprotein, systolic blood pressure, diastolic blood pressure, uric acid, triglyceride, total homocysteine and creatinine*.

**Figure 1:**
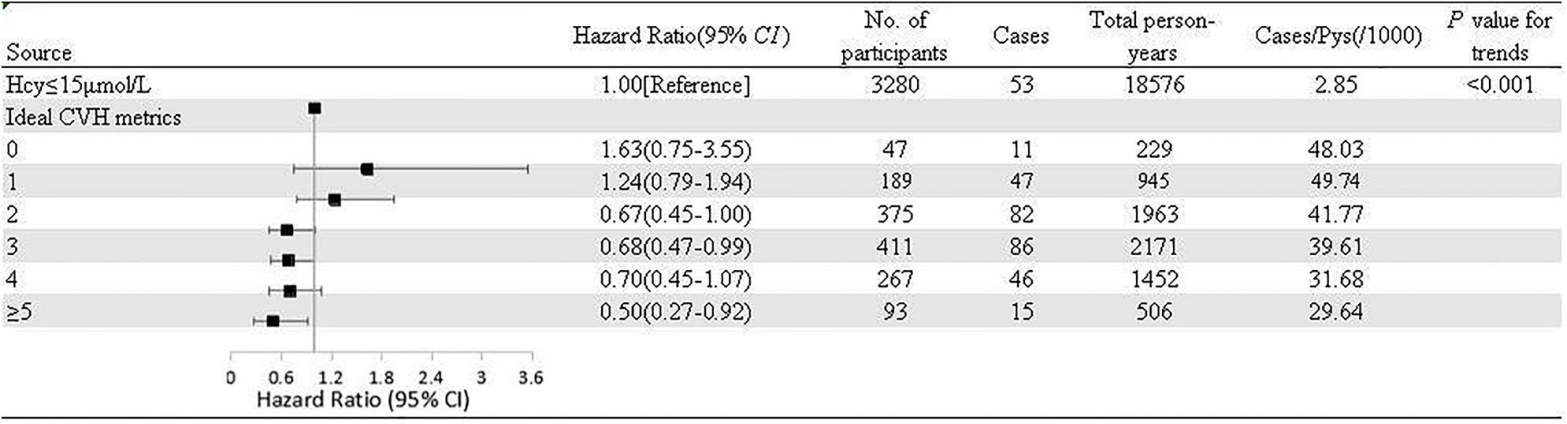
Adjusted hazard ratios and population attributable fractions of ischemic stroke events in relation to number of ideal cardiovascular health metrics among participants with hyperhomocysteinemia compared with normal homocysteine. Pys, Person years; Hcy, homocysteine; CVH, cardiovascular health metrics.

The HR (95% CI) of incident IS risk in relation to the number of ideal CVH metrics among the participants with HHcy, compared with those with normal tHcy levels, is presented in Figure 1. After being adjusted for the aforementioned covariates, the HR (95% CI) of incident risk of IS was 0.50 (0.27–0.92) for the participants with HHcy who had 5–6 CVH metrics, compared with the participants with normal tHcy levels.

### Test for Interactions

Sensitivity analysis was performed for age (<60 years and ≥60 years), hypertension duration (<5 years and ≥5 years), controlled BP (SBP < 130 mmHg or DBP < 80 mmHg and SBP ≥ 130 mmHg or DBP ≥ 80 mmHg), and tHcy levels (<15 and ≥15 μmol/L). There were no significant interactions between the number of ideal CVH metrics and these variables in relation to IS risk (Table 3).

**Table 3:** The HRs (95% CIs) of incident ischemic stroke by Cardiovascular Health Metrics and age, hypertension duration, controlled blood pressure, and homocysteine.

Characteristics^*^	**Cardiovascular health metrics**						***P* trend^†^**	***P* for interaction**
	**0**	**1**	**2**	**3**	**4**	**5–6**		
Age, years^‡^								0.506
<60	1.00	0.82 (0.24–2.84)	0.36 (0.10–1.27)	0.46 (0.13–1.56)	0.39 (0.11–1.42)	0.14 (0.03–0.74)	0.020	
≥60	1.00	0.52 (0.23–1.17)	0.33 (0.15–0.71)	0.31 (0.14–0.68)	0.31 (0.13–0.70)	0.35 (0.14–0.89)	0.025	
Hypertension duration, years^‡^								0.578
<5	1.00	1.52 (0.51–4.54)	0.83 (0.30–2.29)	0.56 (0.20–1.57)	0.77 (0.25–2.36)	0.32 (0.09–1.12)	0.009	
≥5	1.00	0.32 (0.13–0.76)	0.18 (0.08–0.41)	0.20 (0.08–0.49)	0.15 (0.06–0.38)	0.17 (0.06–0.53)	0.006	
Controlled blood pressure, mmHg^‡^								0.508
SBP < 130 or DBP < 80	1.00	0.99 (0.34–2.95)	0.51 (0.17–1.48)	0.58 (0.20–1.67)	0.60 (0.20–1.78)	0.41 (0.12–1.38)	0.108	
SBP≥130 or DBP≥80	1.00	0.32 (0.12–0.85)	0.21 (0.08–0.52)	0.18 (0.07–0.48)	0.16 (0.06–0.47)	0.21 (0.07–0.67)	0.007	
Homocysteine, μmol/L^‡^								0.412
<15	1.00	0.21 (0.09–0.49)	0.11 (0.05–0.24)	0.12 (0.05–0.27)	0.11 (0.05–0.26)	0.13 (0.05–0.35)	0.025	
≥15	1.00	1.21 (0.38–3.84)	0.87 (0.28–2.65)	0.65 (0.19–2.15)	0.61 (0.17–2.19)	0.30 (0.06–1.40)	0.012	

*SBP, systolic blood pressure; DBP, diastolic blood pressure*.

* *All non-pregnant parti5cipants older than 20 years with available cardiovascular health metrics were included*.

† *Trends were analyzed by logistic regression model with adjustment for age and sex*.

‡ *Adjusted for age, sex, education, alcohol consumption, diabetes mellitus, depression, family history of stroke, years of hypertension, antihypertensive medication, low density lipoprotein, systolic blood pressure, diastolic blood pressure, uric acid, triglyceride, total homocysteine and creatinne*.

## Discussion

This community-based prospective cohort study revealed that hypertensive patients with a greater number of ideal CVH metrics had a lower incidence of IS during an average follow-up period of 5.7 years. An ideal healthy diet score (7–9 points) and ideal FBG (<5.5 mmol/L) were independently associated with decreased risk of IS. The participants with HHcy who had 5–6 ideal CVH metrics were 50% less likely to develop IS than those with normal tHcy levels.

Consistent with previous studies in China (15, 16), our study found that only 0.75% of all of the subjects met all ideal metrics, and that 26.2% met 4–6 ideal CVH metrics. These findings suggest that the distribution of CVH metrics among hypertensive patients are similar to those in the general population and are not well-controlled. The low prevalence of ideal CVH metrics among the participants in this study might be explained by the relatively ineffective primary care for hypertensive patients provided by CHSCs in Shenzhen (17). Another possible explanation for these findings is the high prevalence of older adults among our participants (49.6% of the participants were >60 years old), the prevalence of healthy behaviors and positive health factors are known to decrease with aging (2). This finding is consistent with previous results showing that the proportion of older participants who met 5 or more ideal CVH metrics was 2–3 times lower than that of younger participants (18).

We noted that the Kaplan-Meier curves for outcomes for metrics 1–3 and 4–6 merged toward the end (at about month 80), which indicated despite the incident risk of IS among participants with 4–6 metrics was lower than those among participants with 1–3 metrics during the most of the time of the follow-up, the incident risk for those with 4–6 metrics were higher at the end of the follow-up.

The prevalence of HHcy in Chinese populations (19), particularly in those with hypertension, is very high (approximately 80.3%) (4), besides, compelling evidence have demonstrated that the risk of IS faced by hypertensive patients with HHcy was 2.18 times higher than that of hypertensives with normal Hcy (3), thus it is very significant to study the CVH based on the stratification of Hcy in China. Importantly, we found that hypertensive patients with HHcy who had 5 or more ideal CVH metrics exhibited a significantly lower risk of IS events than those with normal tHcy levels, which is the major finding of this study. It' s reported that hypertension with HHcy may just result in higher incidence rate of IS events (20), which may explain why hypertensives with HHcy more likely to prevent IS if they had 5–6 ideal CVH metrics, even compared with hypertensives with normal Hcy. Furthermore, another study suggested that lower tHcy levels are significantly associated with a reduction in first stroke risk in Chinese adults with hypertension (21). As a result, in addition to general lifestyle interventions to improve CVH metrics, such as promoting a healthy diet, physical activity, and non-smoking (22), the Hypertension Group, Cardiology Branch of the Chinese Medical Association recommended that supplementation with folic acid, which is abundant in green leafy vegetables and fruits (23), can significantly reduce the risk of first IS among hypertensive patients with HHcy (4). However, in contrast with Western countries, China has not made the folic acid fortification of grain products mandatory, and Chinese populations have a relatively low dietary and supplemental folate intake (24). Therefore, our finding strongly support the promotion of the prevalence of ideal CVH metrics, particularly increasing the intake of vegetables and fruits among hypertensive patients with HHcy in China.

In our study, ideal healthy diet score was significantly associated with lower IS risk among hypertensive patients. Previous studies have indicated that salt reduction (25), intake of fruit and vegetables (5), whole-grain consumption (26), and moderate intake of meat and eggs (26), which make up our ideal healthy diet, were all related to a lower risk of stroke incidence among hypertensive patients. Although the association between Chinese cooking oils (mainly vegetable oils, including rapeseed oil, peanut oil, and soybean oil) (27) and IS among hypertensives is unclear, it is likely that the high omega-6/omega-3 fatty acid ratio in these oils is highly prothrombotic and proinflammatory and contributes to the prevalence of atherosclerosis, diabetes, and obesity (28, 29), which are all risk factors for IS among hypertensives (24, 30). Given that poor eating habits, such as high fat and sucrose intake, insufficient whole-grain consumption, and high salt levels, were still severe among Chinese populations from 1982 to 2012, according to the results of four Chinese national nutrition surveys (31), a healthy diet is essential for Chinese hypertensive patients to reduce the incidence of IS.

The ideal FBG (<5.5 mmol/L) was inversely associated with the risk of IS, which is consistent with prior reports that a baseline FBG concentration of ≥7.0 mmol/L, or diabetes, is a significant and independent predictor of stroke in Chinese hypertensive patients (24). Moreover, the findings of previous studies have demonstrated that diabetes not only independently multiplied the risk of stroke among hypertensives (32), but was itself also an independent risk factor for stroke (33). Therefore, together with existing evidence of the high prevalence of diabetes among Chinese hypertensive patients (34, 35), our findings suggest that the primary mode of preventing IS in Chinese hypertensive patients should be maintaining an ideal FBG.

### Strengths

The strengths of our study include its prospective design, rigorous ascertainment of IS outcomes, and validation of diagnostic methods and biochemical indices at both baseline and at the end of follow-up. To the best of our knowledge, this is also the first study to reveal that the number of ideal CVH metrics is inversely associated with the risk of IS among hypertensive patients. Randomized clinical trials evaluating the effects on IS of multifactorial interventions in hypertensive patients have been sparse and yielded mixed results (23). Observational studies with regard to stroke effects among hypertensives have mainly assessed biochemical indices (36). In this study, taking advantage of the AHA's ideal CVH metrics, we analyzed a comprehensive IS risk factor profile among hypertensive patients to ascertain the association of CVH metrics with IS risk. More importantly, the consideration of the combination of Hcy and hypertension differentiates the current study from other similar studies on ideal CVH.

### Limitations

Nevertheless, several limitations need to be considered. First, the novelty of our study might be limited, as there have been multiple studies about CVH metrics and various vascular diseases risk since 2012 (6, 16, 18), however, considering the higher prevalence of HHcy among hypertensive patients in China, our study have advantages in promoting healthy behaviors in those populations. Second, the sample resource of single center and single district, as well as loss of more than 15% of participants during follow-up might lead to the selection bias, thus the findings of our study should be interpreted with caution. Also, our findings are not generalizable to the general population due to the inclusion of only a subset of the Chinese population from Shenzhen. Besides, because Shenzhen is an immigrant city with a large population mobility, only local residents who had lived in Shenzhen for ≥6 months were included in our study, however, this might lead to selection bias, because the selected population would clearly be different from those who do not have these habitat characteristics. Third, the assessments of diet score, smoking status, and physical activity were based on self-reporting, so the exposure levels of those variables might not completely accurate. Moreover, although we modified definitions of smoking status, BMI, and a healthy diet from the AHA, these metrics may still be less valid and reproducible in Chinese populations. Fourth, the metrics of healthy behavior were evaluated only at baseline, so changes in these metrics over time could not be accounted for in this study. Furthermore, although we adjusted for the fruit intake, age, and SBP metrics that differed significantly between the participants lost and not lost to follow-up in the multivariable regression, there were still follow-up biases. Finally, we could not completely rule out all of the residual confounders that have been shown to be associated with the risk of IS, such as income level (37). Moreover, the use of medications (anti-diabetics, lipid-lowering, aspirin, nitrates) were not available in our study, thus limited the further analysis.

## Conclusions

In this Chinese hypertensive population-based prospective study, we found that the number of ideal CVH metrics was inversely associated with the incidence of IS. Specifically, the participants with HHcy who had five or more ideal CVH metrics exhibited a lower IS risk than the participants with normal tHcy levels. In addition, an ideal healthy diet score (7–9 points) and ideal FBG (<5.5 mmol/L) were significantly related to a lower risk of IS. Our findings provide guidance for promoting healthy behaviors and health factors to prevent IS among populations with hypertension, especially those with HHcy.

## Data Availability Statement

The raw data supporting the conclusions of this article will be made available by the authors on reasonable request, without undue reservation.

## Ethics Statement

The studies involving human participants were reviewed and approved by Ethics Committee of the Shenzhen Nanshan Center for Chronic Disease Control. The patients/participants provided their written informed consent to participate in this study.

## Author Contributions

YY, LH, FK, and SL conceived and designed the study. SL collected the data. LH and CW organized the database. YY and FK executed the statistical analysis and drafted the manuscript. All authors contributed to manuscript revision, read, and approved the submitted version.

## Conflict of Interest

The authors declare that the research was conducted in the absence of any commercial or financial relationships that could be construed as a potential conflict of interest.

## References

[1] Wang LD Liu JM Yang Y Peng B Wang YL. The prevention and treatment of stroke still face huge challenges—brief report on stroke prevention and treatment in China, 2018. Chin Circ J. (2019) 34:105–19. 10.3969/j.issn.1000-3614.2019.02.001

[2] Mozaffarian D Benjamin EJ Go AS Arnett DK Blaha MJ Cushman M. Heart disease and stroke statistics-2016 update: a report from the american heart association. Circulation. (2016) 133:e38–360. 10.1161/CIR.0000000000000350 26673558

[3] Han LY Wu QH Wang CY Hao YH Zhao JS Zhang LN. Homocysteine, ischemic stroke, and coronary heart disease in hypertensive patients: a population-based, prospective cohort study. Stroke. (2015) 46:1777–86. 10.1161/STROKEAHA.115.009111 26038522

[4] Huo Y Li J Qin X Huang Y Wang X Gottesman RF. Efficacy of folic acid therapy in primary prevention of stroke among adults with hypertension in China: the CSPPT randomized clinical trial. JAMA. (2015) 313:1325–35. 10.1001/jama.2015.2274 25771069

[5] Li JP Jiang SQ Zhang Y Tang G Wang Y Mao G. H-type hypertension and risk of stroke in chinese adults: a prospective, nested case-control study. J Trans Intern Med. (2015) 3:171–8. 10.1515/jtim-2015-0027 27847909 PMC4936453

[6] Dong C Rundek T Wright CB Anwar Z Elkind MS Sacco RL. Ideal cardiovascular health predicts lower risks of myocardial infarction, stroke, and vascular death across whites, blacks, and hispanics: the northern Manhattan study. Circulation. (2012) 125:2975–84. 10.1161/CIRCULATIONAHA.111.081083 22619283 PMC3396556

[7] Bundy JD Ning H Zhong VW Paluch AE Lloyd-Jones DM Wilkins JT. Cardiovascular health score and lifetime risk of cardiovascular disease: the cardiovascular lifetime risk pooling project. Circ Cardiovasc Qual Outcomes. (2020). [Epub ahead of print]. 10.1161/CIRCOUTCOMES.119.006450. 32600064 PMC7772276

[8] Mancia G De Backer G Dominiczak A Cifkova R Fagard R Germano G. 2007 Guidelines for the management of arterial hypertension: the Task Force for the Management of Arterial Hypertension of the European Society of Hypertension (ESH) and of the European Society of Cardiology (ESC). Eur Heart J. (2007) 28:1462–536. 10.1097/HJH.0b013e3282f0580f 17562668

[9] Writing Group of 2018. Chinese Guidelines for the Management of Hypertension. Chin J Cardiovasc Med. (2019) 24:24–56. 10.3969/j.issn.1007-5410.2019.01.002

[10] Lloyd-Jones DM Hong Y Labarthe D Mozaffarian D Appel LJ Van Horn L. Defining and setting national goals for cardiovascular health promotion and disease reduction: the American Heart Association's strategic Impact Goal through 2020 and beyond. Circulation. (2010) 121:586–613. 10.1161/CIRCULATIONAHA.109.192703 20089546

[11] Stroke−1989. Recommendations on stroke prevention, diagnosis, and therapy. report of the WHO Task Force on stroke and other cerebrovascular disorders. Stroke. (1989) 20:1407–31. 10.1161/01.STR.20.10.1407 2799873

[12] Freiberg JJ Tybjaerg-Hansen A Jensen JS Nordestgaard BG. Nonfasting triglycerides and risk of ischemic stroke in the general population. JAMA. (2008) 300:2142–52. 10.1001/jama.2008.621 19001625

[13] Sacco RL Kasner SE Broderick JP Caplan LR Connors JJ Culebras A. An updated definition of stroke for the 21st century: a statement for healthcare professionals from the American Heart Association/American Stroke Association. Stroke. (2013) 44:2064–89. 10.1161/STR.0b013e318296aeca 23652265 PMC11078537

[14] Adams HP Bendixen BH Kappelle LJ Biller J Love BB Gordon DL. Classification of subtype of acute ischemic stroke. definitions for use in a multicenter clinical trial. TOAST. Trial of Org 10172 in Acute Stroke Treatment. Stroke. (1993) 24:35–41. 10.1161/01.STR.24.1.35 7678184

[15] Wu HY Sun ZH Cao DP Wu LX Zeng Q. Cardiovascular health status in Chinese adults in urban areas: analysis of the Chinese Health Examination Database 2010. Int J Cardiol. (2013) 168:760–4. 10.1016/j.ijcard.2012.09.235 23103145

[16] Lu Y Shen S Qi H Fang N Li F Wang L. Prevalence of ideal cardiovascular health in southeast Chinese adults. Int J Cardiol. (2015) 184:385–7. 10.1016/j.ijcard.2015.02.104 25745989

[17] Li H Wei X Wong MC Wong SY Yang N Griffiths SM. A cross-sectional comparison of perceived quality of primary care by hypertensive patients in shanghai and shenzhen, China. Medicine. (2015) 94:e1388. 10.1097/MD.0000000000001388 26313780 PMC4602902

[18] Yang Q Cogswell ME Flanders WD Hong Y Zhang Z Loustalot F. Trends in cardiovascular health metrics and associations with all-cause and CVD mortality among US adults. JAMA. (2012) 307:1273–83. 10.1001/jama.2012.339 22427615 PMC9004324

[19] Yang B Fan S Zhi X Wang Y Wang Y Zheng Q. Prevalence of hyperhomocysteinemia in China: a systematic review and meta-analysis. Nutrients. (2014) 7:74–90. 10.3390/nu7010074 25551247 PMC4303827

[20] Li T Zhu J Fang Q Duan X Zhang M Diao S. Association of H-type hypertension with stroke severity and prognosis. Biomed Res Int. (2018) 2018:8725908. 10.1155/2018/8725908 30271787 PMC6151242

[21] Huang X Li Y Li P Li J Bao H Zhang Y. Association between percent decline in serum total homocysteine and risk of first stroke. Neurology. (2017) 89:2101–7. 10.1212/WNL.0000000000004648 29030456

[22] Hua K Hao G Li W. Cardiovascular outcomes of lifestyle intervention in hypertensive patients with antihypertensive agents. Int J Cardiol. (2017) 227:751–6. 10.1016/j.ijcard.2016.10.062 27810294

[23] Rathor L Akhoon BA Pandey S Srivastava S Pandey R. Folic acid supplementation at lower doses increases oxidative stress resistance and longevity in Caenorhabditis elegans. Age. (2015) 37:113. 10.1007/s11357-015-9850-5 26546011 PMC5005867

[24] Xu RB Kong X Xu BP Song Y Ji M Zhao M. Longitudinal association between fasting blood glucose concentrations and first stroke in hypertensive adults in China: effect of folic acid intervention. Am J Clin Nutr. (2017) 105:564–70. 10.3945/ajcn.116.145656 28122783

[25] Appel LJ. Another major role for dietary sodium reduction: improving blood pressure control in patients with resistant hypertension. Hypertension. (2009) 54:444–6. 10.1161/HYPERTENSIONAHA.109.132944 19620515

[26] De Pergola G D'Alessandro A. Influence of mediterranean diet on blood pressure. Nutrients. (2018) 10:1700. 10.3390/nu10111700 30405063 PMC6266047

[27] Monitoring Report on Nutrition Health Status of Chinese Residents (2010-2013). (2019). Available online at: http://www.docin.com/p-1480413826.html (accessed April 12, 2020).

[28] Haque M Rahman M Alam M Akter S. A possible approach for maintaining effective omega-6/omega-3 fatty acid ratio from mixed vegetable oils. J Environ Sci Natl Res. (2017) 9:65–9. 10.3329/jesnr.v9i2.32159

[29] Simopoulos AP. An increase in the omega-6/omega-3 fatty acid ratio increases the risk for obesity. Nutrients. (2016) 8:128. 10.3390/nu8030128 26950145 PMC4808858

[30] Qureshi AI Caplan LR. Intracranial atherosclerosis. Lancet. (2014) 383:984–98. 10.1016/S0140-6736(13)61088-0 24007975

[31] Chinese Center for Disease Control and Prevention. Report on Nutrition and Chronic Diseases of Chinese Residents. Beijing: People's Health Publishing House (2015). p. 225.

[32] Owolabi MO Agunloye AM. Risk factors for stroke among patients with hypertension: a case-control study. J Neurol Sci. (2013) 325:51–6. 10.1016/j.jns.2012.11.016 23260320

[33] O'Donnell MJ Chin SL Rangarajan S Xavier D Liu L Zhang H. Global and regional effects of potentially modifiable risk factors associated with acute stroke in 32 countries (INTERSTROKE): a case-control study. Lancet. (2016) 388:761–75. 10.1016/S0140-6736(16)30506-2 27431356

[34] Yu S Sun Z Zheng L Guo X Yang H Sun Y. Prevalence of diabetes and impaired fasting glucose in hypertensive adults in rural china: far from leveling-Off. Int J Environ Res Public Health. (2015) 12:14764–79. 10.3390/ijerph121114764 26610531 PMC4661678

[35] Qin X Li J Zhang Y Ma W Fan F Wang B. Prevalence and associated factors of diabetes and impaired fasting glucose in Chinese hypertensive adults aged 45 to 75 years. PLoS ONE. (2012) 7:e42538. 10.1371/journal.pone.0042538 22880024 PMC3411819

[36] Sun P Liu L Liu C Zhang Y Yang Y Qin X. Carotid intima-media thickness and the risk of first stroke in patients with hypertension. Stroke. (2020) 51:379–86. 10.1161/STROKEAHA.119.026587 31948356

[37] Eshak ES Honjo K Iso H Ikeda A Inoue M Sawada Net. Changes in the employment status and risk of stroke and stroke types. Stroke. (2017) 48:1176–82. 10.1161/STROKEAHA.117.016967 28386039

