# Corrigendum: Ideal Cardiovascular Health Metrics and Incidence of Ischemic Stroke Among Hypertensive Patients: A Prospective Cohort Study

**DOI:** 10.3389/fcvm.2021.829078

**Published:** 2021-12-21

**Authors:** Yuchen Ying, Shaoyi Lin, Fanqian Kong, Yuying Li, Shujun Xu, Xiaofeng Liang, Changyi Wang, Liyuan Han

**Affiliations:** ^1^Zhejiang Provincial Key Laboratory of Pathophysiology, School of Medicine, Ningbo University, Ningbo, China; ^2^Department of Elderly Health Care and Management, School of Health Services and Management, Ningbo College of Health Sciences, Ningbo, China; ^3^Cardiology Department, Ningbo First Hospital, Ningbo, China; ^4^Department of Medical Record and Statistics, Ningbo Medical Center Lihuili Hospital, Ningbo, China; ^5^Shenzhen Polytechnic, Shenzhen, China; ^6^Chinese Preventive Medicine Association, Beijing, China; ^7^Department of Chronic Disease Prevention and Control, Shenzhen Nanshan Center for Chronic Disease Control, Shenzhen, China; ^8^Hwa Mei Hospital, University of Chinese Academy of Sciences, Ningbo, China; ^9^Department of Global Health, Ningbo Institute of Life and Health Industry, University of Chinese Academy of Sciences, Ningbo, China

**Keywords:** ideal cardiovascular health metrics, ischemic stroke, hypertensive patients, prospective study, hyperhomocysteinemia

In the original article, we neglected to include the funder Zhejiang Province Public Welfare Technology Application Research Project, Grant No. LGF21H090004 to Yuchen Ying, the funder Medical and Health Science and Technology Plan Project of Zhejiang Province, Grant No. 2021KY335 to Yuchen Ying, and the funder Science and Technology Innovation Activity Plan of Zhejiang University Student & XinMiao Talents Program, Grant No. 2021R464003 to Yuchen Ying. The updated funding statement appears below:

This work was supported by the National Key R&D Program of China [Grant Nos. 2017YFC1310902 and 2018YFC1315305]; Ningbo Health Branding Subject Fund [Grant Nos. PPXK2018-01 and PPXK2018-02]; Sanming Project of Medicine in Shenzhen [Grant No. SZSM201803080]; The Special Innovation Program (Natural Science) of Guangdong Education Department [Grant No. 2018GKTSCX077]; and Natural Science Foundation of Zhejiang Province [Grant No. LQ20H020001], Zhejiang Province Public Welfare Technology Application Research Project [Grant No. LGF21H090004] to YY, Medical and Health Science and Technology Plan Project of Zhejiang Province [Grant No. 2021KY335] to YY, and Science and Technology Innovation Activity Plan of Zhejiang University Student & XinMiao Talents Program [Grant No. 2021R464003] to YY. The funding body had no further role in the study design, the collection, analysis, and interpretation of data, the writing of the manuscript and the decision to submit the paper for publication.

In the published article, there was also an error regarding the affiliation(s) for Yuchen Ying. As well as having affiliation 1, they should also have Department of Elderly Health Care and Management, School of Health Services and Management, Ningbo College of Health Sciences, Ningbo, China.

The authors apologize for these errors and state that this does not change the scientific conclusions of the article in any way. The original article has been updated.

## Publisher's Note

All claims expressed in this article are solely those of the authors and do not necessarily represent those of their affiliated organizations, or those of the publisher, the editors and the reviewers. Any product that may be evaluated in this article, or claim that may be made by its manufacturer, is not guaranteed or endorsed by the publisher.

